# Adoption, Use, and Impact of E-Booking in Private Medical Practices: Mixed-Methods Evaluation of a Two-Year Showcase Project in Canada

**DOI:** 10.2196/medinform.3669

**Published:** 2014-09-24

**Authors:** Guy Paré, Marie-Claude Trudel, Pascal Forget

**Affiliations:** ^1^HEC MontrealMontreal, QCCanada; ^2^Université du Québec à Trois-RivièresTrois-Rivières, QCCanada

**Keywords:** e-booking, medical practices, primary care, missed appointments, mixed-methods evaluative study

## Abstract

**Background:**

Managing appointments in private medical practices and ambulatory care settings is a complex process. Various strategies to reduce missed appointments can be implemented. E-booking systems, which allow patients to schedule and manage medical appointments online, represents such a strategy. To better support clinicians seeking to offer an e-booking service to their patients, health authorities in Canada recently invested in a showcase project involving six private medical clinics.

**Objective:**

The objectives pursued in this study were threefold: (1) to measure adoption and use of the e-booking system in each of the clinics over a 2-year period, (2) to assess patients’ perceptions regarding the characteristics and benefits of using the system, and (3) to measure the impact of the e-booking system on the number of missed appointments in each clinic.

**Methods:**

A mixed-methods approach was adopted in this study. We first extracted and analyzed raw data from the e-booking system deployed in each of the medical practices to monitor adoption and use of the system over time and to assess the impact of the system on the number of missed appointments. Second, we conducted a Web-based survey of patients’ perceptions in the spring of 2013.

**Results:**

The patients and physicians targeted by this showcase project showed a growing interest in the e-booking system as the number of users, time slots made available by physicians, and online appointments grew steadily over time. The great majority of patients said that they appreciated the system mainly because of the benefits they derived from it, namely, scheduling flexibility, time savings, and automated reminders that prevented forgotten appointments. Importantly, our findings suggest that the system’s automated reminders help significantly reduce the number of missed appointments.

**Conclusions:**

E-booking systems seem to represent a win-win solution for patients and physicians in private medical practices. We encourage researchers to replicate and extend our work in other primary care settings in order to test the generalizability of our findings.

## Introduction

One of the keys to efficiency, productivity, and profitability in private medical practices is linked to the appointment scheduling system. Managing appointments in private medical practices and ambulatory care settings is a complex process. One frequent problem faced by many clinics is related to non-attendance [1]. According to various studies, missed appointments (also called “no-shows”) represent close to 10% of all medical appointments [2,3]. There are many collateral effects associated with missed appointments for the providers, staff, and the patients themselves. For instance, no-shows can lead to lower productivity for family physicians and their staff [4]. More importantly, missed appointments increase overall wait time for all patients and can lead to additional risks to their health condition [3].

Various strategies to reduce missed appointments can be found in the extant literature [5]. One frequently mentioned approach is overscheduling, which consists of booking more appointments than the practice is actually able to accommodate [6]. While this strategy may be efficient from the standpoint of use of staff time, it usually creates a great deal of dissatisfaction for both patients and staff [7]. Another approach involves reminders, which are sent in various ways, such as by mail, telephone calls (automated or not), emails, and text messages. These are intended to minimize the risk of patients forgetting their appointments. Several studies have compared the impact of various communication methods for sending out reminders. For example, Henderson [3] observed a decrease in missed appointments when telephone or mailed reminders were used, especially when these reminders were made a few days before the appointment date. Others have observed that text message reminders are as effective as other types [8-10].

Another strategy is called advanced access scheduling [11,12]. This involves reserving appointment slots for same-day appointments, rather than booking appointment slots months in advance. In other words, physicians who use advanced access scheduling generally cut down on prescheduled visits, leaving a large portion of their day open for same-day visits. The mix between prescheduled and open appointments is usually determined by the medical practice’s unique balance of supply and demand for appointments. Research has indicated that advanced access scheduling can provide numerous benefits, including increased satisfaction for patients, providers, and staff [13], fewer missed appointments [14,15], as well as increased productivity among the health care professionals [13].

E-booking systems, which allow patients to schedule and manage their medical appointments online, have also been deployed to streamline management of appointments in medical practices and ambulatory care settings [16,17]. While only 7% of Canadian family physicians (compared to 30% in the United States and 51% in Norway) offered such access in 2012 [18], 90% of surveyed Canadians in 2013 said that if the functionality were available, they would be likely to book an appointment with their health care provider electronically [19]. Survey respondents also ranked e-booking in the top three most useful online consumer health services, just behind electronic prescription renewals and viewing their lab results online. That said, when asked whether they can currently make an appointment with their family physician electronically, only 5% responded that they could.

To better support clinicians seeking to offer an e-booking service to their patients, Canada Health Infoway, a federally funded, not-for-profit organization tasked with accelerating the development of health information technologies across Canada, recently launched the *e-Booking Initiative* for eligible licensed physicians in private medical practices. This program offers financial support to help offset the costs associated with e-booking system acquisition and implementation. Canada Health Infoway also invested in a showcase project involving six private medical clinics located in Québec, Canada. The present study pursued three objectives in line with this multisite project: (1) to measure adoption (number of patients and physicians enrolled) and use (number of time slots available online, number of appointments made online) of the e-booking system in each of the clinics over a 2-year period, that is, between January 2012 and December 2013; (2) to assess patients’ perceptions regarding the characteristics and benefits of the e-booking system; and (3) to measure the impact of system usage on the number of missed appointments in each participating clinic. Evidence for effective technological solutions to streamline the appointment scheduling process and improve attendance in primary care and outpatient settings is lacking. Indeed, very few empirical studies [20] have investigated the adoption, use, and effectiveness of e-booking systems in private medical practices. Hence, the present study attempts to fill this gap.

## Methods

### E-Booking System and Sites

The *Doctor Direct* software application (DoctorDirect.com) was deployed as part of this showcase project. This application consists of a secure Web portal that enables patients to access their doctor’s schedule 24 hours a day, 7 days a week and book an appointment that suits them best without the assistance of a secretary. An email reminder, as well as a telephone reminder (automated message), are sent to the patient 2 days before the appointment. The patient is then able to confirm or cancel the appointment online. This solution was chosen because of its interoperability with the most widely used electronic medical record (EMR) system (Kinlogix Medical, TELUS Health) in medical practices in Québec [21]. The medical practices that took part in this project (see Table 1) were identified by Canada Health Infoway; they were chosen mainly because of the diversity of their profiles in terms of health care services offered and clients. Acronyms have been used to preserve anonymity of the participating clinics. It was decided that each medical practice would adopt a marketing strategy to promote the e-appointment system with its clients. As shown later, the promotion strategy for each medical practice was developed based on the patients’ sociodemographic characteristics and level of comfort with the technology, as well as the preferred methods of promotion identified by the management at each site. Medical practices did not receive any financial incentives to encourage participation in this showcase project.

**Table 1:** Profile of the medical practices.

Medical practice	Health care services offered	Clients
A	Family medicine with two specialists on-site	Adults and children
B	Family medicine, travel health, specimen collection center, operating rooms	Adults and children
C	Transrectal echography with or without biopsy, cystoscopy, vasectomy, uroflowmetry, minor surgery, urology research	Elderly clients / primarily men
D	Medical consultation with or without an appointment, emergency and minor surgery, specimen collection service for laboratory testing, mother-child clinic, vaccination	Young families / expectant women or mothers with babies
E	General medicine	Ubisoft employees (young computer-savvy people)
F	Multidisciplinary health services	Mostly adults or elderly people

### Data Collection and Analysis

A mixed-methods approach was adopted in this study. First, to monitor adoption and use of the e-booking system over the 2-year observation period, the supplier of the IT solution gave us secure access to the system’s database. This allowed us to extract raw usage data from the e-booking system in use at each of the six medical practices. These data were then imported into an Excel file that was used to produce several graphs (see Results section). In line with our second objective, a Web-based questionnaire survey was conducted in the spring of 2013. Of the 4338 patients enrolled in the e-booking system at the start of the study, 1032 (23.79%) agreed to be contacted by the research team. The questionnaire, which was prepared in French and English, was posted online using *Qualtrics* software and an email invitation to take part in the study was sent to all potential respondents. A week later, an email reminder was sent to all targeted respondents. As shown in the next section, data were analyzed using various descriptive statistics (means, standard deviations) and tests (Pearson’s chi-square test, Student’s *t* test) as well as partial least square (PLS) multiple regression tests.

Our third and final objective was to assess the impact of the use of the e-booking system on the number of missed appointments. To this end, we began by analyzing data from Clinic A, which had recorded the most appointments made online in the period from January 1, 2012 to December 31, 2013. We compiled the number of offline appointments (made through a secretary), the number of online appointments, and the number of missed appointments (offline and online) from January 2012 to November 2013. A statistical *t* test analysis allowed us to measure the impact of the e-booking system on the number of missed appointments. Data were then collected on the four other medical clinics (B, C, D, and E) from the databases of their e-booking systems. Data for a 12-month period (December 2012 to November 2013) were analyzed, since the volume of online appointments was high enough to perform the desired analyses. Data from Clinic F were not analyzed since the volume of online appointments was too low. Data were analyzed using Student’s *t* test.

Ethical approval for this study was obtained from the Research Ethics Council of HEC Montréal in March 2013.

## Results

### Adoption and Use of the E-Booking System

**Figure 1:**
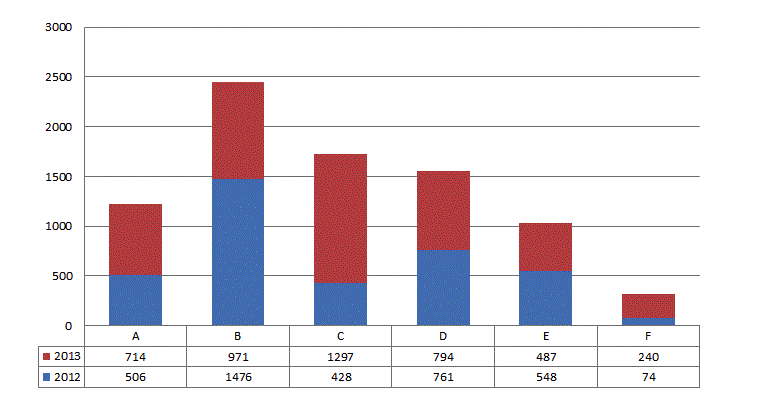
Number of new patients enrolled, by medical practice.

The statistics presented in Figure 1 show that 8296 patients from the six medical practices enrolled with *Doctor Direct.* This represents 10.00% (3793/37,936) and 12.00% (4503/37,524) of the active patients at all six clinics in 2012 and 2013, respectively. Five of the six clinics recruited 1600 new registrants, on average, from the time they deployed the e-booking system to the end of 2013. Clinic F, which had more difficulty recruiting patients to use the system, had only 250 patients registered at the end of 2013. According to those responsible for the project, various technical problems (eg, appointment confirmations not sent, time slots offered to more than one patient), which had occurred mostly in 2012, represented an aggravating factor for this site.

**Figure 2:**
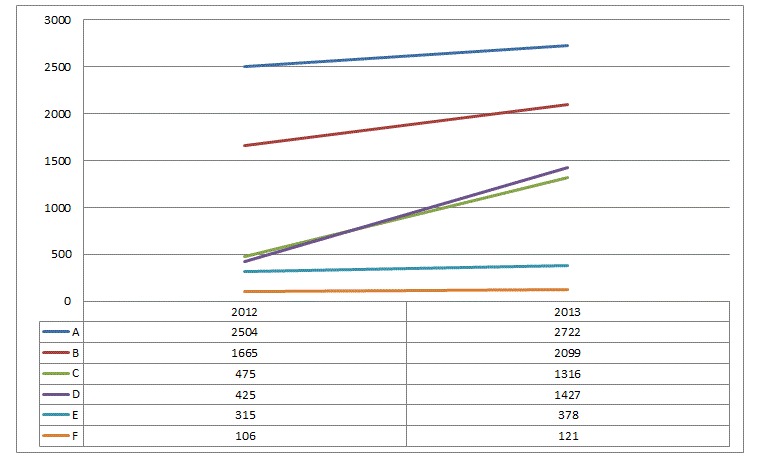
Number of medical appointments booked online in 2012 and 2013, by clinic.

At the end of 2012, there were 34 physicians using the system in six clinics for a total of 50 possible licenses (68%). Twelve months later, 47 licenses (94%) were being used by the targeted physicians. In terms of system use, the number of time slots that the physicians had made available online grew from 23,201 in 2012 to 43,101 in 2013, for a 46% increase. As shown in Figure 2, the number of medical appointments booked online by patients grew by 32%, from 5490 in 2012 to 8063 in 2013, bringing the number of online appointments to 13,553. This represented a total of one out of every five time slots assigned to the online reservation system. Last, the average registered patient made 1.6 online appointments from the time they enrolled in the system until December 31, 2013.

### Survey of Patients Enrolled in the E-Booking System

A total of 228 completed questionnaires were received between March 29 and April 3. As mentioned above, a reminder letter was sent to all targeted respondents on April 4. This reminder helped retrieve an additional 147 questionnaires. The final response rate was 36.34% (375/1032), which is deemed satisfactory [22]. Among the questionnaires received, 71 had to be discarded due to missing data. The final sample was thus comprised of 304 questionnaires, including 194 received before the reminder and 110 after the reminder. As there was no statistically significant difference between early and late respondents on all attributes, response bias was unlikely [23].

As shown in Table 2, the sample consisted of two main categories of respondents: patients who had already made at least one appointment online since enrolling in the e-booking system (n=241) and patients who had not yet made an appointment using the system (n=63). The results show similarities between the two groups as to sex, age, and level of education. The sample included slightly more women than men and all age groups were represented, although individuals aged 50 to 59 years represented the main group of respondents. Four out of five respondents had a college diploma or university degree, which shows a high level of education.

We began by asking patients who had not yet booked an appointment online (n=63) to state their reasons for not doing so. The main reason was that they had not needed to schedule a doctor’s appointment between the time they enrolled and the study period (n=24). However, more than one-third of non-users (33%, 21/63) indicated that they had tried to schedule an appointment but were unable to do so because no time slot was available for their doctor. Technical problems during their first attempt discouraged only 14 respondents. It is worth mentioning that system user-friendliness and security did not seem to be major barriers to system use. We also asked this sub-group of patients the extent to which they intended to schedule their next medical appointments online. About 85% (54/63) responded positively.

We then turned our attention to patients who had booked at least one medical appointment online using *Doctor Direct* (n=241). The majority of system users (56.0%, 135/241) had booked only one appointment online, while one in four (24.0%) had booked two appointments and 20.0% had booked three or more. The vast majority (83.0%, 200/241) used the system to manage their own medical appointments, while only 17.0% (41/241) used it to book appointments for relatives. As shown in Table 3, users of the e-appointment system claimed to be very satisfied (average of 4.2 on a scale of 5), perceived the system as very user-friendly (4.3/5), and had a firm intention of continuing to use it in the future (4.5/5).

**Figure 3:**
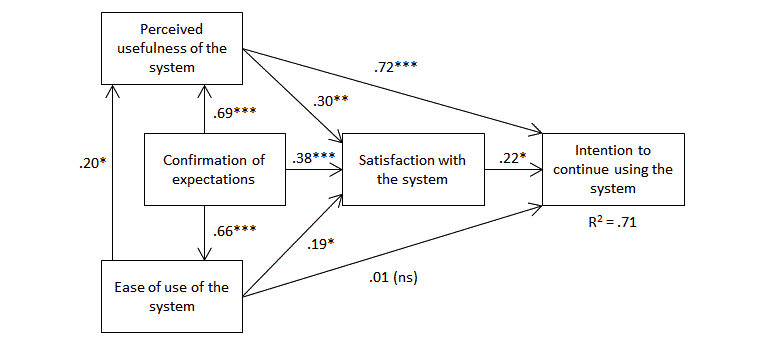
Research model and PLS results (n=241). ***P<.005; **P<.01; *P<.05; ns=not significant.

To further investigate the factors that motivate patients to continue using the e-appointment system in the future, we tested a research model derived from the works of Bhattacherjee [24] and Hong et al [25] on information systems continuance. As shown in Figure 3, our model suggests that an individual’s intention to continue using a computer-based system is mainly influenced by his or her level of satisfaction toward the system. In turn, user satisfaction is influenced by the extent to which initial expectations toward the system are confirmed as well as by two factors from the TAM (technology acceptance model) proposed by Davis [26], namely, system ease of use and system usefulness. Following Hong et al [25], our model also proposes direct links between the TAM constructs and the dependent variable. The survey instrument that was used is presented in Multimedia Appendix 1. The reliability of the measures was determined with Cronbach alpha. Findings in Table 3 indicate that all the measures, without exception, meet or surpass the .70 threshold of statistical significance [27]. This table also demonstrates the validity of the variables included in our research model. In particular, we see that the square root of the variance shared by each variable and its respective items is greater than the inter-correlations between the variables.

PLS regression analyses were performed to test the links in our model. Our findings supported all relationships, with the exception of the association between system ease of use and continuance intention. It would thus appear that system user-friendliness has an indirect effect on the dependent variable via its direct influence on user satisfaction. Most importantly, our findings underline the importance of the “expectation confirmation” variable which, as anticipated, is strongly related to TAM factors and user satisfaction. This result shows the importance of managing users’ initial expectations to ensure that they are not disappointed when they first attempt to use the system.

Next, Table 4 indicates that three kinds of benefits were perceived by system users: scheduling flexibility, time savings, and automated reminders that prevented forgotten appointments.

Concerning the marketing or promotional strategies implemented in each medical clinic, we asked all respondents (n=304) to indicate what had led them to enroll in the e-booking system. As shown in Table 5, half of them mentioned that they enrolled because a secretary had recommended it during a prior visit to the clinic. One out of five patients signed on to the Internet portal at the recommendation of their physician, and approximately 15% were inspired by the message on the clinic’s voicemail and the tab on the medical clinic’s website. The brochures and posters promoting the portal in the clinics’ waiting rooms appeared to have had little effect on enrollments, since they were mentioned by only 6% of respondents. No significant statistical differences were found across medical practices.

**Table 2:** Profile of survey respondents (n=304).

		Patients who booked online at least once (n=241)	Patients yet to book online (n=63)	χ^2^ and *t*	*P* value
		n (%)	n (%)		
**Sex**					
	Men	109 (45.2)	22 (34.9)	χ^2^=1.7	.197
	Women	131 (55.4)	39 (61.9)		
**Age, years**					
	18–29	21 (8.7)	8 (12.7)	χ^2^=1.3	.933
	30–39	60 (24.9)	13 (20.6)		
	40–49	29 (12.0)	8 (12.7)		
	50–59	66 (27.4)	18 (28.6)		
	60–69	46 (19.1)	11 (17.5)		
	70+	19 (7.8)	5 (7.9)		
**Education**					
	None	4 (1.7)	0 (0.0)	χ^2^=6.3	.279
	High school diploma	44 (18.3)	10 (15.9)		
	College diploma	54 (22.4)	16 (25.4)		
	Bachelor degree	73 (30.2)	26 (41.3)		
	Master’s degree	53 (22.0)	7 (11.1)		
	PhD	12 (5.0)	3 (4.8)		
**Medical practices**					
	A	18 (7.5)	6 (9.5)	χ^2^=55.1	.000
	B	70 (29.0)	39 (61.9)		
	C	77 (32.0)	2 (3.2)		
	D	57 (23.7)	7 (11.1)		
	E	13 (5.4)	6 (9.5)		
	F	6 (2.5)	3 (4.8)		
**Level of computer knowledge** ^a^		4.5	4.2	*t*=2.3	.022

^a^Scale of 1 to 5 where 1=slightly familiar and 5=very familiar.

**Table 3:** Descriptive statistics and variance shared by the variables.

	Mean	SD	Number of items	Cronbach alpha	PU	EOU	CONF	SAT	CONT
Perceived usefulness of the system (PU)	4.2	0.9	4	.86	.85 ^a^				
User-friendliness of the system (EOU)	4.3	0.8	4	.93	.68^b^	.91			
Confirmation of expectations (CONF)	4.0	1.0	3	.87	.82^b^	.68^b^	.89		
Satisfaction with the system (SAT)	4.2	0.9	4	.80	.72^b^	.58^b^	.73^b^	.82	
Intention to continue using the system (CONT)	4.5	0.8	3	.93	.81^b^	.62^b^	.76^b^	.72^b^	.94

^a^The ratios on the diagonal represent the square root of the variance shared by each variable and its respective items. The ratios below the diagonal are correlations between variables.

^b^ *P*<.001.

**Table 4:** Perceived benefits of using the e-booking system (n=241).

		Average (1-5 scale)	SD
**Greater flexibility**			
	Makes it possible to book appointments when it is most convenient.	4.7	1.0
	Greater flexibility in the choice of available time slots.	4.6	1.2
**Time savings**			
	Saves time by eliminating waiting on the phone.	4.5	1.0
	Saves time by eliminating the need for reminders several times at the clinic when the phone is busy.	4.5	1.2
	Saves time by eliminating the need for me to go in person to the clinic to schedule an appointment.	4.5	1.2
**Reduction in forgotten appointments**			
	Makes it easier to remember appointments thanks to reminders.	4.5	1.0

**Table 5:** Promotional strategies put in place and patients’ receptiveness (n=304).

Promotional strategy	Clinic A	Clinic B	Clinic C	Clinic D	Clinic E	Clinic F	Patients who were influenced, n (%)
Secretary’s verbal recommendation	√^a^	√	√	√	√	√	158 (52.0)
Physician’s verbal recommendation	√	√	√	√	√	√	62 (20.3)
Promotional message on the clinic’s voicemail	√	√	√	√	√	√	49 (16.1)
Link on the medical clinic’s website		√	√	√		X	45 (14.8)
Flyer distributed at the medical clinic	√	√	√	√	√	√	21 (6.9)
Promotional poster in the medical clinic	√	√	√	√	√	√	17 (5.6)
Interactive terminals available in the clinic (iPads)	X^b^		X	√		X	-
Email invitation to all patients					√	√	N/A^c^

^a^√ = Strategy implemented before the survey conducted in the spring of 2013.

^b^X=Strategy implemented after the survey conducted in the spring of 2013.

^c^N/A=Data not available in the survey questionnaire.

### Impact of E-Booking on the Number of Missed Appointments

**Figure 4:**
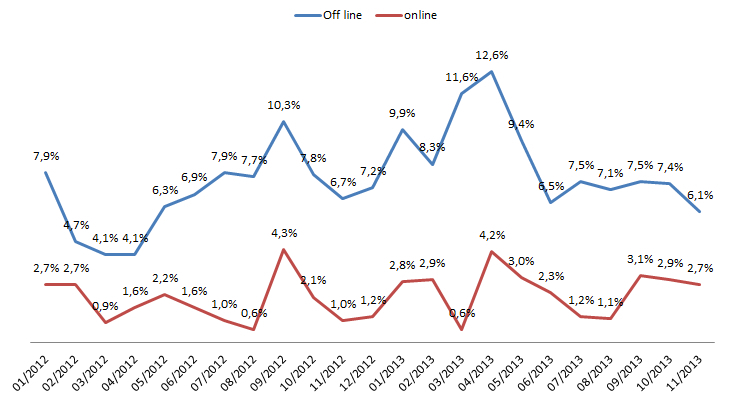
Proportion of missed appointments at medical practice A.

Our third and final goal was to assess the impact of the e-booking system on the number of missed appointments. As explained above, we began by analyzing Clinic A’s data. The results shown in Figure 4 indicate that the percentage of missed appointments each month varied from 3.4% to 11% and averaged 6.5%. However, when we compare appointments made online (by the patients themselves) from those made offline, we note a large difference in the number of missed appointments. The percentage of online appointments that were missed varied from 0.6% to 4.3%, averaging 2.1%. Considering appointments made in the traditional manner, missed appointments represented 4.1% to 12.6% of the total and averaged 7.6%. The difference between the two groups (offline and online) in terms of the number of missed appointments is statistically significant (*t*=8.8; *P*<.001).

Similar results were then obtained from four other medical practices over a 12-month observation period from December 2012 to November 2013: Clinic B (*t*=6.3; *P*<.001), Clinic C (*t*=5.8; *P*<.001), Clinic D (*t*=4.0; *P*<.005), and Clinic E (*t*=2.2; *P*<.05).

## Discussion

### Principal Findings

Overall, the patients targeted by this showcase project showed a growing interest in the e-booking system as the number of users grew steadily over time. The promotion strategies that had greatest impact on the number of enrollments were verbal recommendations from a secretary and, to a lesser extent, from the attending physician. The great majority of users said that they appreciated the system because they found it user-friendly and for the benefits they derived from it, and this can be seen in the constantly increasing number of appointments made online over the 2-year period. Three main categories of benefits were perceived by patients, namely, scheduling flexibility, time savings, and automated reminders that prevented forgotten appointments. Findings also reveal that the number of time slots opened up by the physicians also grew month after month, and this represents a critical success factor [16]. Indeed, those respondents who had tried to schedule an appointment but were unable to do so because no time slot was available for their doctor are among those who had no intention of continuing to use the e-booking in the future. Last, in line with prior findings [20], our study reveals that the use of an e-booking system can help significantly reduce the number of no-shows or missed appointments.

Despite the encouraging results presented above, some physicians were still hesitant to make time slots available online. One reason cited by our respondents was related to the fact that there are different types of medical appointments (eg, routine annual examinations, prenatal check-ups, surgical follow-up), and they vary in length. This constraint was discussed during the project, and a strategy was developed in response: the development of pop-up menus. Such menus act as filters that, through structured questions (eg, the patient’s first appointment: yes/no, a diagnosis requiring follow-up, etc.), lead the patient to select the right type of appointment, that is, one for the right amount of time. In addition to this solution, we believe that better integration of the e-booking system into the EMR system used by each clinic could facilitate the allocation of time slots by adapting the type of time slot to the health condition of each patient. Last, it is important to manage physicians’ expectations. If a physician has not freed up a sufficient number of time slots for online appointments, patients may lose interest and stop using the system. Setting realistic objectives by carefully targeting the percentage of time slots to be offered online and/or by beginning with specific types of appointments (eg, vaccination clinics or short, regular follow-up appointments) may encourage a gradual transition to routine system use.

With regard to promotional strategies, secretaries and physicians must continue to encourage patients to use the e-booking system, particularly since such use leads to a significant decline in missed appointments. It would appear important to emphasize the benefits of system use: flexibility in making appointments, the time saved, and automated reminders, which prevent patients from forgetting their appointments, rather than the system’s features, such as its user-friendliness, security, and reliability. Another suggestion would be to send periodic reminders to patients enrolled in the system so that they will not forget about the system and about having enrolled in it. These reminders should clearly present how to recover forgotten user codes and passwords. To prevent these messages from being perceived as junk mail and ignored, they could be combined with general information designed to make patients more responsible for their health or by public health messages.

### Limitations

The results of this study must be interpreted with caution due to its inherent limitations. For one thing, we are mindful of the small scale of the showcase project. Future studies should try to validate our findings among a larger number of medical practices and contexts. We also recognize the usual constraints and generalization limitations associated with cross-sectional surveys [22]. Next, it is important to mention, with respect to generalization, that our survey was limited, as we were unable to estimate the characteristics of the reference population. This is a direct consequence of using, as a recruitment strategy, voluntary participation for completing an online questionnaire. Importantly, we analyzed secondary and survey data associated with a single e-booking system that necessarily has its own characteristics. Our findings must therefore be replicated with other e-booking platforms. Last, it would also be interesting to carry out in-depth interviews with actual users (both patients and physicians) of e-booking systems so as to gain a richer insight into the data obtained through survey questionnaires.

### Conclusions

In short, the main purpose of this study was to assess perceived and actual outcomes following the deployment of an e-booking system in six medical practices in Canada. Our results show that e-booking systems seem to represent a win-win solution for patients and physicians. For one thing, patients appreciate using such a system due to its flexibility and the fact that use allows them to save time. Further, our analyses suggest that the system’s automated reminders help significantly reduce the number of missed appointments, a problem that plagues several medical practices. We encourage health informatics researchers to replicate and extend our work in other primary care settings in order to test the generalizability of our results.

## Multimedia Appendix 1

Survey instrument.

## Abbreviations

EMR: electronic medical record
PLS: partial least square
TAM: technology acceptance model
