# Supplementary material for: Adoption, Use, and Impact of E-Booking in Private Medical Practices: Mixed-Methods Evaluation of a Two-Year Showcase Project in Canada
Source: JMIR Med Inform. 2014 Sep 24;2(2):e24. doi: 10.2196/medinform.3669 (PMC4288107; doi:10.2196/medinform.3669)
Supplement: Supplementary file 1 [file medinform_v2i2e24_app1.pdf]

## Multimedia Appendix 1. Survey instrument

A. Questions for those who have booked medical appointments online

For each of the following statements, select the item that best fits your personal opinion with the online medical appointment system:

| | Strongly disagree | | Neutral | | Strongly agree |
|---|---|---|---|---|---|
| Using the online medical appointment system helps me book appointments more quickly | 1 | 2 | 3 | 4 | 5 |
| Using the online medical appointment system helps me not forget appointments | 1 | 2 | 3 | 4 | 5 |
| Learning how to use the online medical appointment system is easy | 1 | 2 | 3 | 4 | 5 |
| The online medical appointment system is user-friendly | 1 | 2 | 3 | 4 | 5 |
| My experience with using the online medical appointment system was better than what I expected | 1 | 2 | 3 | 4 | 5 |
| I intend to continue my use of the online medical appointment system in the future | 1 | 2 | 3 | 4 | 5 |
| Using the online medical appointment system makes my life easier | 1 | 2 | 3 | 4 | 5 |
| Using the online medical appointment system has provided me with more benefits than I initially expected | 1 | 2 | 3 | 4 | 5 |
| The online medical appointment system is clear and understandable to use | 1 | 2 | 3 | 4 | 5 |
| I have no intention to stop using the online medical appointment system | 1 | 2 | 3 | 4 | 5 |
| Overall, I find the online medical appointment system useful in my life | 1 | 2 | 3 | 4 | 5 |
| Overall, I find the online medical appointment system easy to use | 1 | 2 | 3 | 4 | 5 |
| Overall, most of my expectations from using the online medical appointment system were confirmed | 1 | 2 | 3 | 4 | 5 |
| My intentions are to continue using the online medical appointment system to book future appointments with my doctor | 1 | 2 | 3 | 4 | 5 |

How do you feel about your overall experience of the online medical appointment system usage?

| | | | | | | |
|---|---|---|---|---|---|---|
| Very dissatisfied | 1 | 2 | 3 | 4 | 5 | Very satisfied |
| Very frustrated | 1 | 2 | 3 | 4 | 5 | Very contented |
| Very displeased | 1 | 2 | 3 | 4 | 5 | Very pleased |
| Absolutely terrible | 1 | 2 | 3 | 4 | 5 | Absolutely delighted |

Indicate for each of the following statements, to what extent the use of the online medical appointment system has provided you with certain benefits:

| | Not at all | | | | A lot | Not applicable |
|---|---|---|---|---|---|---|
| Saves time by eliminating wait time on the phone | 1 | 2 | 3 | 4 | 5 | |
| Saves time by eliminating the need to redial the clinic's number over and over when the line is busy or when no one answers | 1 | 2 | 3 | 4 | 5 | |
| Saves time by eliminating the need to go in person to the clinic to make an appointment | 1 | 2 | 3 | 4 | 5 | |
| Greater flexibility in the choice of time slots because I can see online all the doctor's availability | 1 | 2 | 3 | 4 | 5 | |
| Possibility to make an appointment at a time that suits me best even when the clinic is closed (24 hours, 7 days a week) | 1 | 2 | 3 | 4 | 5 | |
| Avoid forgetting an appointment through automated reminders (6) | 1 | 2 | 3 | 4 | 5 | |

What factor(s) motivated you to register online with the online medical appointment system (check all that apply)

- ❑ Recommendation or suggestion from the medical clinic's secretary
- ❑ Recommendation or suggestion from the doctor
- ❑ Brochure or business card provided at the medical clinic
- ❑ Clinic's advertising poster
- ❑ Medical clinic's website
- ❑ Medical clinic's voicemail greeting message
- ❑ Other : ____________________

B. Questions for those who have not booked any medical appointments online

Please check the main reason(s) why you haven't booked any medical appointments online since you first registered with the online medical appointment system? (Check all that apply)

- ❑ Since I registered with the online medical appointment system I have not had to book any medical appointments
- ❑ I tried to book a medical appointment online but I encountered some technical problems with the system
- ❑ I tried to book a medical appointment online but there was no available slot for my doctor
- ❑ The online medical appointment system is too complicated for me
- ❑ The interface of the online medical appointment system is not user-friendly
- ❑ The online medical appointment system is not secure enough
- ❑ I fear for my privacy
- ❑ I simply prefer to continue booking medical appointments as before
- ❑ Other : ____________________

For each statement below, select the answer that best fits your personal opinion.

| | **Strongly disagree** | | **Neutral** | | **Strongly agree** |
|---|---|---|---|---|---|
| I intend to book future medical appointments (mine or my close ones') online using the online medical appointment system | 1 | 2 | 3 | 4 | 5 |
| I do not wish to invest time in learning how to use the online medical appointment system | 1 | 2 | 3 | 4 | 5 |
| I will consider using the online medical appointment system to book future medical appointments | 1 | 2 | 3 | 4 | 5 |

What factor(s) motivated you to register online with the online medical appointment system (check all that apply)

- ❑ Recommendation or suggestion from the medical clinic's secretary
- ❑ Recommendation or suggestion from the doctor
- ❑ Brochure or business card provided at the medical clinic
- ❑ Clinic's advertising poster
- ❑ Medical clinic's website
- ❑ Medical clinic's voicemail greeting message
- ❑ Other : ____________________
